# Assessment of Estrogenic and Genotoxic Activity in Wastewater Using Planar Bioassays

**DOI:** 10.3390/toxics13110936

**Published:** 2025-10-30

**Authors:** Markus Windisch, Valentina Rieser, Clemens Kittinger

**Affiliations:** Diagnostic and Research Institute of Hygiene, Microbiology and Environmental Medicine, Diagnostic and Research Center for Molecular Biomedicine, Medical University of Graz, 8010 Graz, Austria; markus.windisch@medunigraz.at (M.W.);

**Keywords:** high-performance thin-layer chromatography, wastewater, estrogenicity, genotoxicity, effect-directed analysis

## Abstract

The contamination of ground and surface waters with micropollutants like estrogenic compounds and genotoxins is a major public health concern. Conventional wastewater treatment plants are currently not capable of completely removing those contaminants. In this study, we applied planar bioassays to investigate the genotoxicity and estrogenic activity of hospital and municipal wastewater from an Austrian treatment plant. Using the open-source 2LabsToGo platform in combination with the HPTLC-SOS-UmuC and HPTLC-YES assays, both genotoxic and estrogenic compound zones were detected in untreated wastewater. Genotoxic activity was found in sewage sludge filtrate and hospital wastewater, with bioanalytical concentrations ranging from 1.6 to 21.8 µg 4-NQO-EQ L^−1^. Estrogenic responses were observed in the influent and hospital wastewater samples, with BEQ values between 3.5 and 16.0 µg E2-EQ L^−1^. No activity was detected in the treated effluent, indicating efficient removal of these compounds during wastewater treatment. These results confirm the presence of biologically active micropollutants in hospitals and raw wastewater and demonstrate the suitability of planar bioassays for sensitive, spatially resolved detection. The use of portable equipment like the 2LabsToGo system suggests that on-site monitoring of estrogenic and genotoxic activities in wastewater is feasible and could support routine surveillance of treatment efficiency.

## 1. Introduction

Micropollutants in wastewater have emerged as a critical environmental and public health concern due to their persistence, bioactivity, and potential to disrupt ecosystems. In this context, estrogenic compounds (ECs) and genotoxins are of particular concern, as they can have significant biological effects even at trace levels. ECs, which include natural estrogens (e.g., estrone and estradiol), synthetic pharmaceuticals (e.g., ethinylestradiol in oral contraceptives), and industrial chemicals (e.g., alkylphenols and bisphenol A), are frequently detected in wastewater effluents [1,2]. Their presence poses significant risks to aquatic organisms and, potentially, to human health, as they can interfere with the endocrine systems of exposed organisms. Since conventional wastewater treatment plants are not designed to fully eliminate estrogens, they may be released and accumulate in the environment [2]. Numerous studies have demonstrated the biological effects of estrogenic compounds on aquatic organisms [3,4,5]. Exposure of male fish to ECs, for example, has been shown to induce synthesis of the egg-yolk protein vitellogenin, usually only produced by female fish [6]. A study by Purdom et al. found an over 1000-fold increase in plasma vitellogenin when male trout were maintained in effluent water for three weeks [7]. Alongside the negative consequences for aquatic environments, in humans, chronic exposure to exogenous ECs has been linked to adverse health outcomes. Studies have associated environmental estrogens with an increased risk of hormone-dependent cancers, such as breast and ovarian cancer in women [8,9], as well as testicular cancer and reduced sperm quality in men [10]. Additionally, ECs have been implicated in developmental and reproductive disorders, including early puberty in women and reduced fertility in both sexes [11]. Among several potential routes of exposure to environmental ECs, ingestion of contaminated drinking water is of particular concern. Even though, in most countries, the concentration of ECs in drinking water is generally below thresholds that would suggest an immediate effect on the endocrine system, concerns have been raised about chronic low-level exposure [12]. This highlights the importance of thorough removal of ECs from wastewater to prevent contamination of ground and surface water bodies that may act as drinking water sources.

Alongside ECs, genotoxins are frequently detected in wastewater and its associated matrices [13]. Similar to estrogens, the release of genotoxins into the environment can pose risks to aquatic organisms by inducing genomic alterations that may impair reproduction, development, and population stability [14]. In this context, hospital wastewater is of particular concern as it contains residues from various pharmaceuticals, including antibiotics, cytostatics, or even radionuclides from cancer therapy [15]. Thus, hospital wastewater is frequently tested positive for genotoxicity [16,17,18].

The complexity of wastewater matrices, combined with the persistence and bioactivity of ECs and genotoxins, presents significant analytical challenges. The current state-of-the-art approach involves advanced chromatographic techniques coupled with mass spectrometry, such as liquid chromatography–tandem mass spectrometry (LC-MS/MS) and gas chromatography–mass spectrometry (GC-MS). These methods offer high sensitivity, specificity, and the ability to simultaneously quantify multiple compounds [19]. However, they often require sample preparation, including solid-phase extraction (SPE) or liquid–liquid extraction (LLE), to concentrate analytes and remove interfering substances [20]. This can be time-consuming and costly, particularly when analyzing large numbers of samples. Additionally, the presence of complex organic and inorganic constituents in wastewater can lead to matrix effects, such as ion suppression or enhancement, which can compromise the accuracy and reliability of results [21]. Furthermore, analytical chemistry alone gives no information on the biological effects of the detected substances. Since wastewater is a complex matrix with potentially thousands of substances, synergistic or antagonistic toxicological effects can be overlooked by screening for single compounds. In this context, in vitro biological assays, or short bioassays, offer a valuable addition, as they provide a cumulative response for a particular biological endpoint (e.g., genotoxicity or endocrine activity). Genotoxicity of water and wastewater is commonly assessed with the UmuC assay, which has been codified under ISO 13829, while endocrine activity can be analyzed using the Yeast Estrogen/Androgen Screen (YES/YAS) assay. While these assays have been used in different studies to determine genotoxicity and endocrine activity of sludge and wastewater samples [22], they still have limitations. Commonly, in vitro bioassays are performed using agar or microtiter plates. To ensure adequate bacterial growth, a certain dilution of the culture medium or agar must not be exceeded, which, in turn, limits the amount of sample that can be used. Therefore, native water samples are rarely utilized, and sample preparation steps like pre-concentration or extraction are necessary. These preparatory steps, however, carry the risk of excluding potentially important contaminants, which consequently may be overlooked by the analysis. Furthermore, the sensitivity of conventional in vitro bioassays is generally not sufficient to comply with proposed European environmental quality standards [23]. In this context, planar bioassays offer a valuable alternative. These assays combine thin-layer chromatography (TLC) with in vitro bioassays. The chromatographic separation prior to the bioassay performance has been demonstrated to increase sensitivity while reducing potential matrix effects. Furthermore, planar bioassays have been successfully validated against their conventional counterparts [24]. The combination of TLC with the YES assay has been successfully utilized for the screening of estrogenic compounds in a variety of consumer products like tomatoes and cosmetics [25,26]. In the case of water and wastewater, the planar YES and YAS assays have been used to detect compounds with endocrine activity in samples from different wastewater treatment plants [27]. While the results of these investigations proved the applicability of planar bioassays for effect-directed analysis in complex matrices like wastewater, sample preparation and extraction were mandatory.

In the present study, the genotoxicity and endocrine activity of wastewater samples were assessed using planar bioassays. The samples were collected from the sewer system of an Austrian hospital and from the wastewater treatment plant Gössendorf, which treats the municipal wastewater of the city of Graz (Styria, Austria). In contrast to earlier studies, the samples were analyzed natively without any sample preparation, such as extraction or purification. In order to achieve this, the 2LabsToGo open-source system was utilized, which combines a chemical and a biological laboratory in one instrument [28]. The system is equipped with an integrated autosampler with a spraying nozzle for sample applications and a TLC plate heater. Between each spraying cycle, the sample is dried on a preheated TLC plate, which allows the application of large sample volumes. Because the samples are concentrated directly on the plate, surface pre-concentration becomes obsolete. Especially for samples with moderately volatile solvents that have an extended drying time under atmospheric pressure and room temperature (e.g., water), this function proves useful.

## 2. Materials and Methods

### 2.1. Wastewater Samples

Hospital wastewater samples were collected from the sewer system of an Austrian hospital at five different sites. Communal wastewater samples were collected at the wastewater treatment plant in Gössendorf (Styria, Austria) at multiple sampling sites representing different treatment stages: influent A, influent B, primary clarifier, secondary clarifier, sewage sludge filtrate, and effluent.

### 2.2. Chemicals and Materials

The UmuC tester strain *Salmonella* Typhimurium TA 1535 containing the plasmid pSK 1002 was purchased from DSMZ German Collection of Microorganisms and Cell Cultures (Braunschweig, Germany). The genetically modified *Saccharomyces cerevisiae* RMY326/hERα (YES) strain was purchased from Xenometrix (Allschwil, Switzerland). Sodium dihydrogenphosphate monohydrate (>98%), magnesium sulfate heptahydrate (99.5%), sodium hydroxide (>99%), resorufin-β-d-galactopyranoside, 4-nitroquinoline-1-oxide (4-NQO), HPTLC Silica gel 60 glass plates without F254 and nylon membrane filters (0.22 µm pore size) were purchased from Merck (Darmstadt, Germany). Ampicillin sodium salt (>99%), potassium chloride (≥99.5%) and potassium dihydrogen phosphate (≥99%) were purchased from Carl Roth (Karlsruhe, Germany). Nutrient Broth No. 2 was purchased from Oxoid (Basingstoke, Hampshire, UK). Yeast nitrogen base, L-adenine, L-arginine, L-aspartic acid, L-glutamic acid, L-histidine, L-isoleucine, L-leucine, L-lysine, L-methionine, L-phenylalanine, L-serine, L-threonine, L-tyrosine, L-valine, and copper (II) sulfate were purchased from Merck (Darmstadt, Germany).

### 2.3. Sample Preparation, Buffer, Substrate, Ampicillin and Positive Control Solution

Wastewater samples were membrane filtrated (0.22 µm pore size) before use to remove particulate matter and to prevent clogging of the 2LabsToGo tube system. The retained material was not analyzed further; however, based on visual inspection, the amount was low, and no biological activity is expected to be associated with the removed fraction. A stock solution of resorufin-β-d-galactopyranoside (20 mg/mL) in dimethyl sulfoxide was prepared. Before use, 12.5 µL was dissolved in 2.5 mL phosphate buffer prepared from magnesium sulfate heptahydrate (0.12 g), potassium chloride (0.37 g), potassium dihydrogen phosphate (4.1 g), and disodium phosphate (4.3 g) in 100 mL distilled water. The phosphate buffer was adjusted to pH 7 using solid sodium hydroxide. As positive controls for the bioassays, 17β-estradiol (E2) and 4-nitroquinoline-1-oxide were dissolved in methanol (1 ng/µL). Ampicillin stock solution was prepared from 50 mg ampicillin sodium salt dissolved in 1 mL of deionized water.

### 2.4. Preparation of Bacterial and Yeast Cultures

*Salmonella* Typhimurium TA 1535, pSK 1002 culture was grown overnight in 10 mL of Nutrient Broth No. 2 supplemented with 10 µL of ampicillin solution at 37 °C and 120 rpm shaking. Only cultures that reached an OD_600_ value of at least 2 were used for subsequent experiments. *Saccharomyces cerevisiae* RMY326/hERα culture was prepared in accordance with the instructions provided by the manufacturer. Briefly, a filter disc containing the *S. cerevisiae* strain was added to 5 mL yeast growth medium supplemented with essential amino acids in a vented T25 flask and incubated in an orbital shaker at 31 °C and 75 rpm shaking for 3 days. The dense culture was mixed 1:1 with glycerol (30% in deionized water). Aliquots were stored in cryovials at −80 °C until use. On the day before the experiment, a *Saccharomyces cerevisiae* cryostock (1 mL) was diluted in 10 mL yeast growth medium and incubated for 16 h at 31 °C and 75 rpm shaking.

### 2.5. Workflow of the Planar SOS-Umu-C Genotoxicity Assays

HPTLC glass plates were prewashed with methanol and dried under a fume hood before use. Wastewater samples were applied onto the plate using the autosampler function of the 2LabsToGo system. The applied sample volumes ranged between 50 µL and 80 µL per band. Each TLC plate was heated to 90 °C during sample application, and an interrupted dosing mode with a 12 s pause between each spraying cycle was chosen. The distance between bands was 8 mm, and the distance from the lower and left edge was 10 mm. Subsequently the plates were developed with 3 mL cyclohexane–ethanol 17:3 or ethylacetate–toluene–methanol–water 65:20:11:4, all *v*/*v*, in a 10 cm × 10 cm twin-trough chamber (CAMAG, Switzerland) up to a migration distance of 70 mm. After plate drying for 5 min using a hairdryer, the chromatograms were detected under UV light at 254 nm.

1 µL 4-nitroquinoline-1-oxide (1 ng/µL) was applied as a positive control on the upper part of the plate above the solvent front. The *Salmonella* Typhimurium overnight culture was diluted in fresh Nutrient Broth No. 2 medium to an OD_600_ of 0.2. Afterward, 2.5 mL of cell suspension was piezoelectrically sprayed onto the plate (Derivatizer, level 4, red nozzle, CAMAG, Muttenz, Switzerland). The wet plate was placed horizontally in a humid box and incubated for 3 h at 37 °C. Afterward, the plate was dried for 5 min (hairdryer), and 2.5 mL β-d-galactopyranoside substrate solution was piezoelectrically sprayed onto the plate (Derivatizer, level 4, yellow nozzle, CAMAG, Muttenz, Switzerland). The wet plate was incubated for 45 min at 37 °C, dried (hairdryer), and detected under UV light at 254 nm. Photographs were taken using a smartphone camera (iPhone SE 2020, Apple Inc., Cupertino, CA, USA).

### 2.6. Workflow of the Planar Yeast Estrogen Screen (YES) Assay

Preparation of HPTLC plates, sample application, and TLC workflow were performed as described before. 1 µL of 17β-estradiol (1 ng/µL) was applied as a positive control on the upper part of the plate. The overnight yeast culture was centrifuged at 300× *g* for 5 min, and the cell pellet was resuspended in fresh growth medium supplemented with 150 µM copper (II) sulfate. 2.8 mL cell suspension (8 × 10^7^ cells) was piezoelectrically sprayed onto the plate (CAMAG Derivatizer, level 4, red nozzle). The wet plate was placed horizontally in a humid box and incubated for 3 h at 31 °C. Afterward, the plate was dried for 5 min (hairdryer), and 2.5 mL β-d-galactopyranoside substrate solution was piezoelectrically sprayed onto the plate (CAMAG Derivatizer, level 4, yellow nozzle). The wet plate was incubated for 45 min at 37 °C, dried (hairdryer), and detected under UV light at 254 nm. Photographs were taken using a smartphone camera (iPhone SE 2020, Apple Inc., Cupertino, CA, USA).

### 2.7. Calculation of Reference Compound Biological Equivalent Concentration

HPLTC-bioautograms were analyzed via videodensitometry using the open-source software quanTLC [29]. Semi-quantification was conducted by evaluating the effect produced by a given sample relative to the effect produced by a known control substance. The raw data from the videodensitometric analysis can be found in the Supplemental Material (File S1). The calculation is based on Equation (1) and considers the effect strength as the area under the curve (AUC), the mass (m_control_) of control, and the sample volume (v_sample_) applied. The calculation of the biological equivalence concentrations (BEQ) was based on the reference AUC of 1 ng 4-NQO or 1 ng E2, respectively.

(1) $$BEQ=\frac{{AUC}_{sample}}{{AUC}_{control}}\times \frac{m_{control}}{V_{sample}}$$

## 3. Results

A fast and efficient workflow for non-targeted screening for genotoxic and estrogenic compounds in wastewater was developed. By using the open-source 2LabsToGo system, large sample volumes can be applied directly on the HPTLC surface without the need for sample preparation. This allows for unbiased analysis, minimizing potential loss of toxicologically relevant contaminants. Samples that showed active response zones in the initial planar bioassay screening were reanalyzed in a second, independent experiment on a new HPTLC plate to confirm reproducibility.

### 3.1. Screening for Genotoxins in Communal Wastewater with the Planar SOS-Umu-C Assay

**Figure 1:**
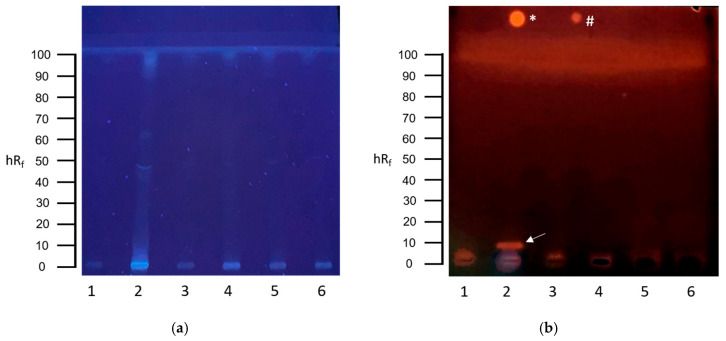
HPTLC-SOS-UmuC assay results of communal wastewater samples. (**a**) HPTLC chromatogram after development with cyclohexane–ethanol 17:3 at UV 254 nm. (**b**) HPTLC bioautogram at UV 254 nm after conducting the SOS-UmuC bioassay. A total of 80 µL of each wastewater sample was applied: (1) effluent, (2) sewage sludge filtrate, (3) secondary clarifier, (4) primary clarifier, (5) influent B, and (6) influent A. Positive response zones are indicated by arrows. Positive controls of 4-nitroquinoline-1-oxide (4-NQO) were applied above the solvent front: * = 10 ng 4-NQO, # = 1 ng 4-NQO.

The planar SOS-Umu-C assay revealed a genotoxic signal in the sewage sludge filtrate as an orange, fluorescent zone at an hR_f_ value of 10 (Figure 1b, track 2).

**Figure 2:**
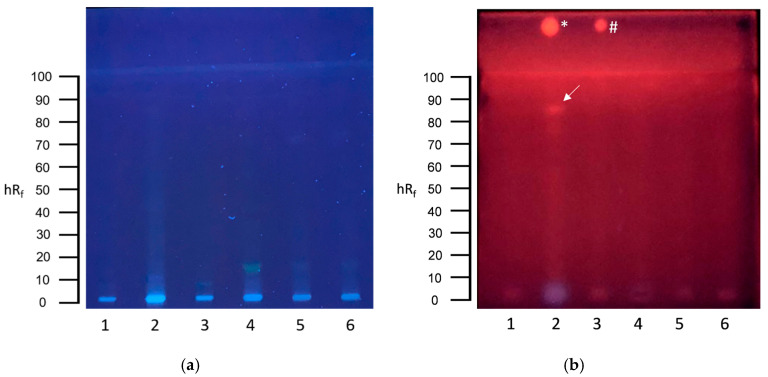
HPTLC-SOS-UmuC assay results of communal wastewater samples. (**a**) HPTLC chromatogram after development with ethylacetate–toluene–methanol–water 65:20:11:4 at UV 254 nm. (**b**) HPTLC bioautogram at UV 254 nm after conducting the SOS-UmuC bioassay. A total of 80 µL of each wastewater sample was applied: (1) effluent, (2) sewage sludge filtrate, (3) secondary clarifier, (4) primary clarifier, (5) influent B, and (6) influent A. Positive response zones are indicated by arrows. Positive controls of 4-nitroquinoline-1-oxide (4-NQO) were applied above the solvent front: * = 10 ng 4-NQO, # = 1 ng 4-NQO.

To achieve better elution of the genotoxic zone, a more polar mobile phase consisting of ethyl-acetate–toluene–methanol–water 65:20:11:4 was utilized for subsequent experiments. This led to a larger migration distance of the genotoxic substance response up to an hRf value of approximately 85 (Figure 2b, track 2). The mean calculated BEQ was 5.6 µg 4-NQO-EQ L^−1^ (range: 1.6–9.6 µg L^−1^, n = 2).

### 3.2. Screening for Genotoxins in Hospital Wastewater with the Planar SOS-Umu-C Assay

**Figure 3:**
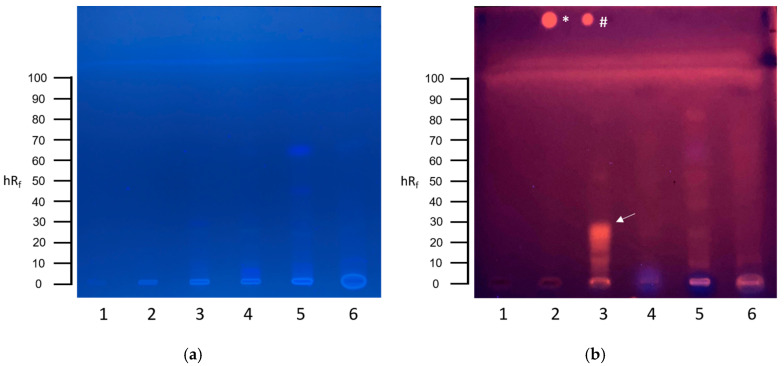
HPTLC-SOS-UmuC assay results of hospital wastewater samples. (**a**) HPTLC chromatogram after development with ethylacetate–toluene–methanol–water 65:20:11:4 at UV 254 nm. (**b**) HPTLC bioautogram at UV 254 nm after conducting the SOS-UmuC bioassay. A total of 50 µL of each wastewater sample was applied: (1) tap water and (2–6) sampling sites of the hospital sewer system. Positive response zones are indicated by arrows. Positive controls of 4-nitroquinoline-1-oxide (4-NQO) were applied above the solvent front: * = 10 ng 4-NQO, # = 1 ng 4-NQO.

The planar SOS-Umu-C assay revealed a genotoxic signal in the hospital wastewater (sampling site 3), visible as an orange, fluorescent zone up to an hRf value of 30 (Figure 3b, track 3). The mean calculated BEQ was 17.5 µg 4-NQO-EQ L^−1^ (range: 13.2–21.8 µg L^−1^, n = 2).

### 3.3. Screening for Estrogenic Substances in Communal Wastewater with the Planar YES Assay

**Figure 4:**
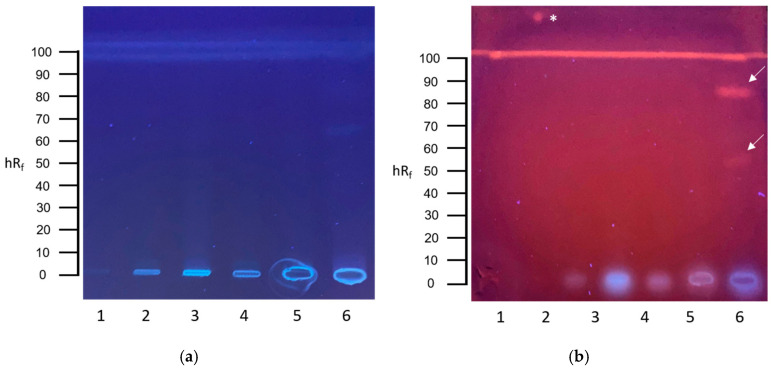
HPTLC-YES assay results of communal wastewater samples. (**a**) HPTLC chromatogram after development with ethylacetate–toluene–methanol–water 65:20:11:4 at UV 254 nm. (**b**) HPTLC bioautogram at UV 254 nm after conducting the YES bioassay. A total of 50 µL of each wastewater sample was applied: (1) tap water, (2) effluent, (3) sewage sludge filtrate, (4) secondary clarifier, (5) primary clarifier, and (6) influent. Positive response zones are indicated by arrows. Positive control of E2 was applied above the solvent front. * = 1 ng E2.

Wastewater screening with the HPTLC-YES assay revealed the presence of estrogenic compounds. Two estrogenic substance responses at hR_f_ 55 and 85 were visible in the influent sample of the wastewater treatment plant (Figure 4b, track 6). The mean calculated BEQ was 7.1 µg E2-EQ L^−1^ (range: 5.2–9.1 µg L^−1^, n = 2).

### 3.4. Screening for Estrogenic Substances in Hospital Wastewater with the Planar YES Assay

**Figure 5:**
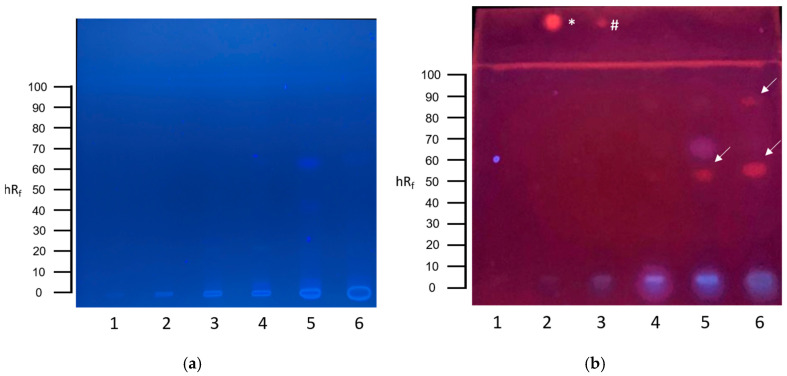
HPTLC-YES assay results of hospital wastewater samples. (**a**) HPTLC chromatogram after development with ethylacetate–toluene–methanol–water 65:20:11:4 at UV 254 nm. (**b**) HPTLC bioautogram at UV 254 nm after conducting the YES bioassay. A total of 50 µL of each hospital wastewater sample was applied: (1) tap water and (2–6) sampling sites of the hospital sewer system. Positive response zones are indicated by arrows. Positive controls of E2 were applied above the solvent front: * = 10 ng E2, # = 1 ng E2.

Screening for estrogenic compounds in hospital wastewater with the HPTLC-YES assay revealed the presence of estrogenic compounds in three sampling sites of the hospital sewer system. Two estrogenic substance responses at approximately hR_f_ 52 and hR_f_ 85 were visible, respectively (Figure 5b, tracks 4, 5, 6). The mean calculated BEQs were 12.6 µg E2-EQ L^−1^ (range: 9.1–16.0 µg L^−1^, n = 2) (track 6, band at hR_f_ 52) and 4.1 µg E2-EQ L^−1^ (range: 3.5–4.6 µg L^−1^, n = 2) (track 6, band at hR_f_ 85).

## 4. Discussion

The results of this study highlight the presence of genotoxic and estrogenic compounds in communal and hospital wastewater, underscoring the environmental and public health concerns associated with the wastewater treatment process. The HPTLC-SOS-UmuC assay revealed a response zone in sewage sludge filtrate and in a hospital wastewater sample, suggesting contamination with genotoxic compounds. This finding is in line with the results of earlier studies that showed the genotoxic potential of sewage sludge and hospital wastewater [13,17]. The positive sample from the wastewater treatment plant consisted of the liquid phase of the dewatered sludge after anaerobic fermentation, which at this point contains high amounts of ammonia and nitrogen. While nitrosamines, which are known genotoxins [30], may form under these conditions, their detection in the HPTLC-SOS-UmuC assay is unlikely without an exogenous metabolic activation system (S9 fraction), as the assay cannot activate such promutagens. Therefore, the observed genotoxicity is more likely attributable to compounds that can be activated intracellularly, such as nitroaromatic substances [31], which could explain the positive signals in the assay. Additionally, a variety of potential genotoxic contaminants, including industrial chemicals, pharmaceuticals, and their transformation products, can accumulate in the sludge during the wastewater treatment process. BEQ values for genotoxicity ranged between 1.6 and 21.8 µg 4-NQO-EQ L^−1^, with hospital wastewater exhibiting higher genotoxic activity compared to communal wastewater. 

The HPTLC-YES assay revealed two estrogenic substance responses in the influent of the wastewater treatment plant. This is expected, as untreated wastewater can contain a variety of estrogenic compounds, ranging from natural to synthetic estrogens as well as industrial chemicals like bisphenol A. In none of the subsequent treatment stages was a positive estrogenic substance response detectable. This may be due to biodegradation of estrogenic compounds during treatment or a transfer from the liquid phase (wastewater) into the solid phase (sludge). The results of this study confirm the findings of earlier investigations, which have shown increased partitioning of estrogens to activated sludge and a decrease in estrogenic activity along the treatment process [19,32]. In the case of hospital wastewater, estrogenic substance responses were detected in three samples. The estrogenic activity could be explained by the presence of natural or synthetic steroid hormones (e.g., estradiol, ethinylestradiol), which are excreted. In addition, pharmaceuticals with endocrine activity may also be present at significant concentrations in hospital wastewater. The BEQ values for estrogenicity ranged between 3.5 and 16.0 µg E2-EQ L^−1^.

Overall, the outcome of this study is positive. The absence of genotoxic and estrogenic signals in the effluent of the wastewater treatment plant suggests an effective removal of these micropollutants during the treatment process. It is worth mentioning, however, that genotoxic and estrogenic compounds may concentrate in the sewage sludge. This can be a cause for concern when sludge is used as a fertilizer in agriculture, which is a common practice in many countries. In Austria, however, since 2023, sewage sludge from wastewater treatment plants with capacities for over 20,000 inhabitants has to be treated through thermal processes, such as incineration. This inactivates most organic pollutants and prevents reintroduction into the environment.

## 5. Conclusions

The method presented in this study enables fast detection of genotoxic and estrogenic compounds in wastewater samples. By using the autosampler function of the 2LabsToGo system with the integrated plate heating function, wastewater samples were directly applied onto the HPTLC plate without any sample preparation besides filtration. While the heating step is technically required for efficient sample application, it cannot be excluded that it may influence the composition or biological activity of some compounds. Future studies should address this aspect. The method presented could be particularly relevant for the routine analysis of wastewater, which is already required regularly for other parameters, such as inorganic phosphorus or nitrogen. It could be proposed that the surveillance of wastewater could be extended by measuring estrogenic or genotoxic activity regularly on a weekly or biweekly schedule.

## Data Availability

The data will be made available upon request which should be sent to markus.windisch@medunigraz.at and are subject to approval by all the named authors participating in this study.

## Supplementary Materials

The following supporting information can be downloaded at https://www.mdpi.com/article/10.3390/toxics13110936/s1. File S1: Compiled raw data of videodensitometric analysis of bioautograms.

## Abbreviations

The following abbreviations are used in this manuscript:

AUC	Area under the curve
BEQ	Biological equivalence concentration
E2	17β-estradiol
ECs	Estrogenic compounds
GC-MS	Gas chromatography–mass spectrometry
HPTLC	High-performance thin-layer chromatography
LC-MS	Liquid chromatography–mass spectrometry
LLE	Liquid–liquid extraction
SPE	Solid-phase extraction
TLC	Thin layer chromatography
YAS	Yeast androgen screen
YES	Yeast estrogen screen
4-NQO	4-nitroquinoline-1-oxide
