# Supplementary material for: Assessment of Estrogenic and Genotoxic Activity in Wastewater Using Planar Bioassays
Source: Toxics. 2025 Oct 30;13(11):936. doi: 10.3390/toxics13110936 (PMC12656073; doi:10.3390/toxics13110936)
Supplement: Supplementary file 1 [file toxics-13-00936-s001.zip › File S1.pdf]

File S1 – Compiled raw data of videodensitometric analysis of bioautograms

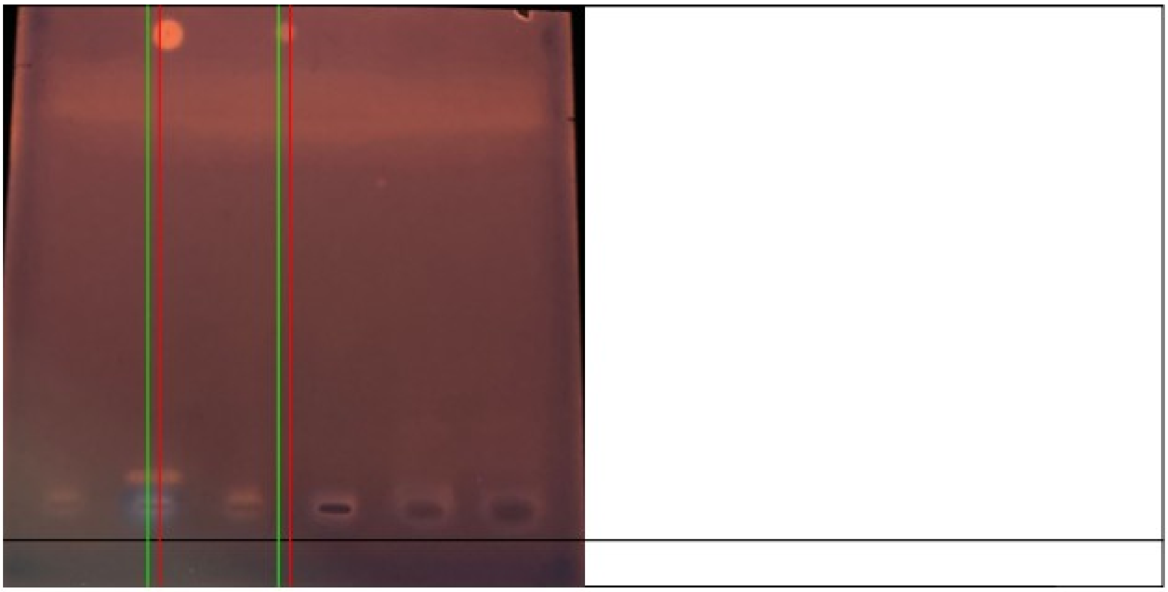

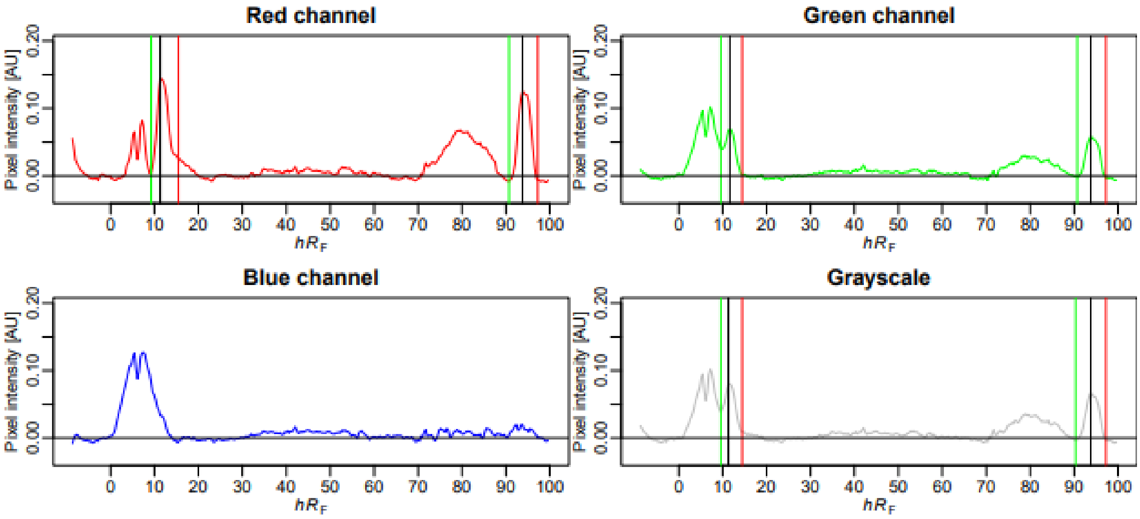

Track	Channel	Start hRf	End hRf	hRf	Height	Area
1	red	91	98	94	0.1264499	1.1352774
1	red	9	16	11	0.1447132	1.3488794
1	green	91	98	94	0.0588352	0.5523888
1	green	10	14	12	0.0692512	0.6146948
1	gray	91	98	94	0.0679330	0.6266336
1	gray	10	14	11	0.0804501	0.7461422

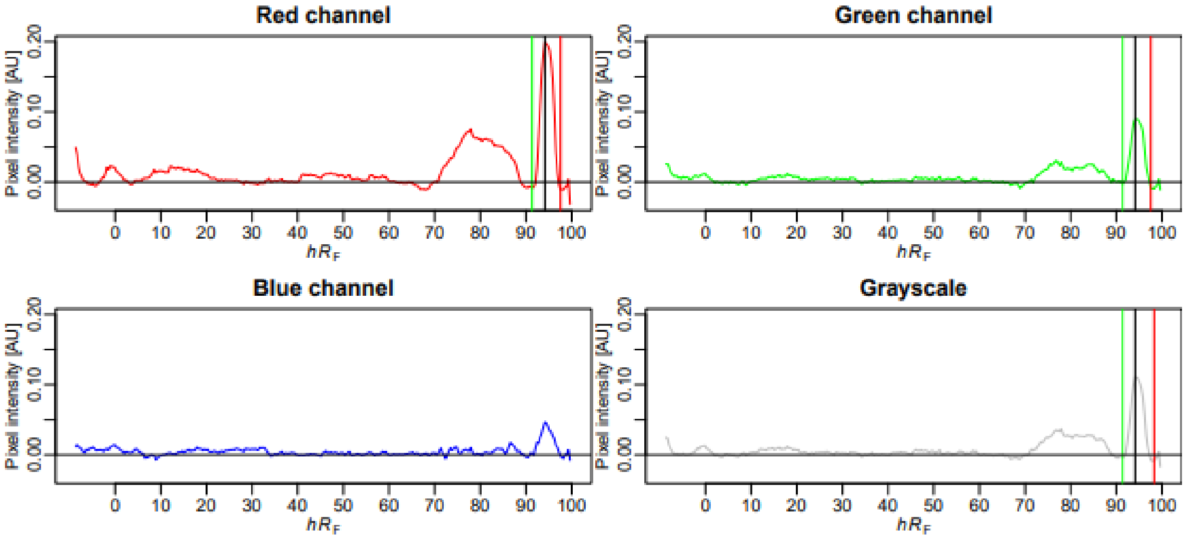

Track	Channel	Start hRf	End hRf	hRf	Height	Area
2	red	92	98	94	0.1985958	1.7481933
2	green	92	98	94	0.0898733	0.8201114
2	gray	92	99	94	0.1111764	0.9713612

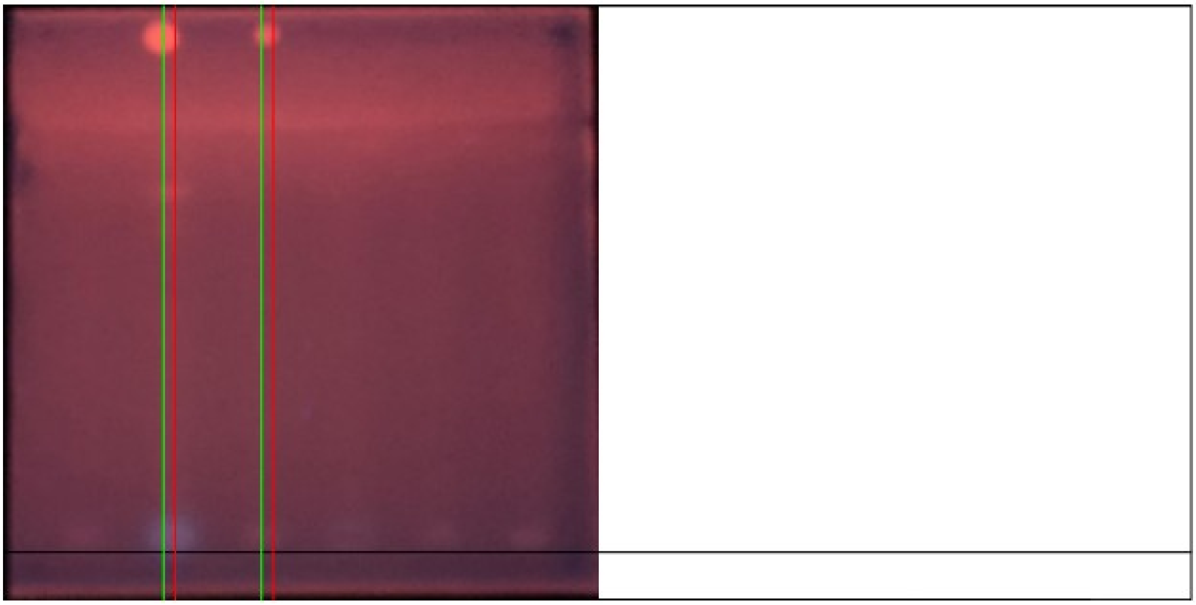

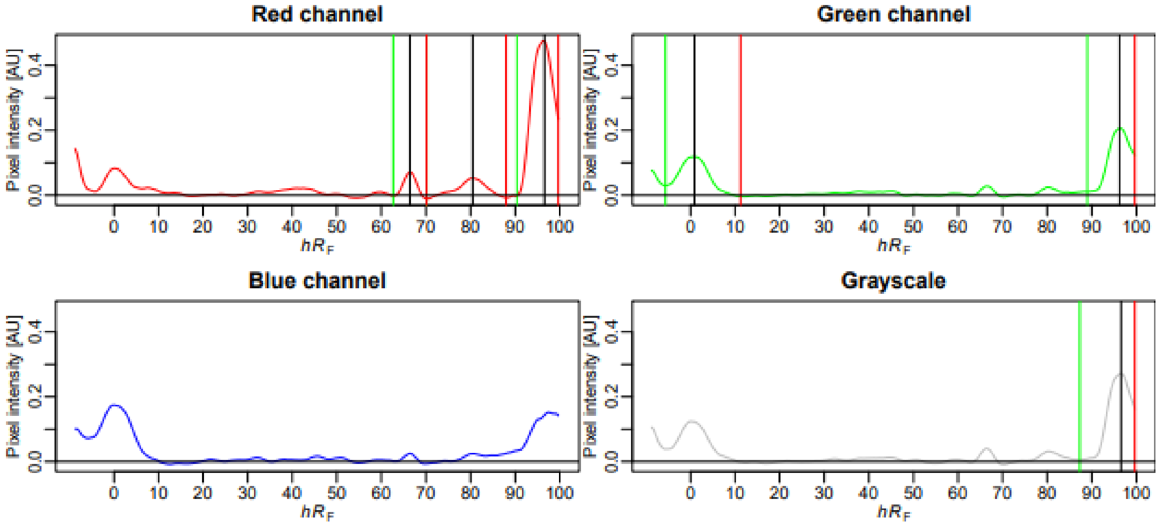

Track	Channel	Start hRf	End hRf	hRf	Height	Area
1	red	91	100	97	0.4753327	8.0963040
1	red	70	88	81	0.0525340	0.9199115
1	red	63	70	67	0.0716647	0.6127742
1	green	89	100	97	0.2066349	3.5804382
1	green	-6	11	1	0.1187462	2.7293086
1	gray	88	100	97	0.2722051	4.8764272

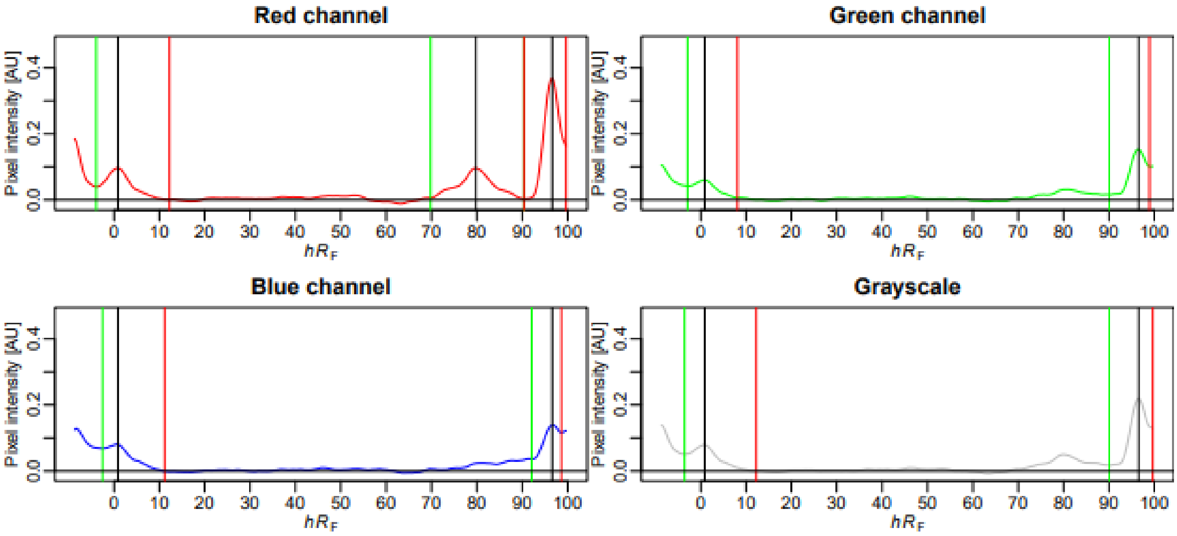

Track	Channel	Start hRf	End hRf	hRf	Height	Area
2	red	91	100	97	0.3679283	4.646973
2	red	70	91	80	0.0961614	2.526830
2	red	-4	12	1	0.0962176	1.966025
2	green	90	99	97	0.1531647	2.097342
2	green	-3	8	1	0.0604676	1.166357
2	blue	92	99	97	0.1393031	1.909318
2	blue	-3	11	1	0.0808160	1.657576
2	gray	90	100	97	0.2201845	3.144387
2	gray	-4	12	1	0.0792251	1.694287

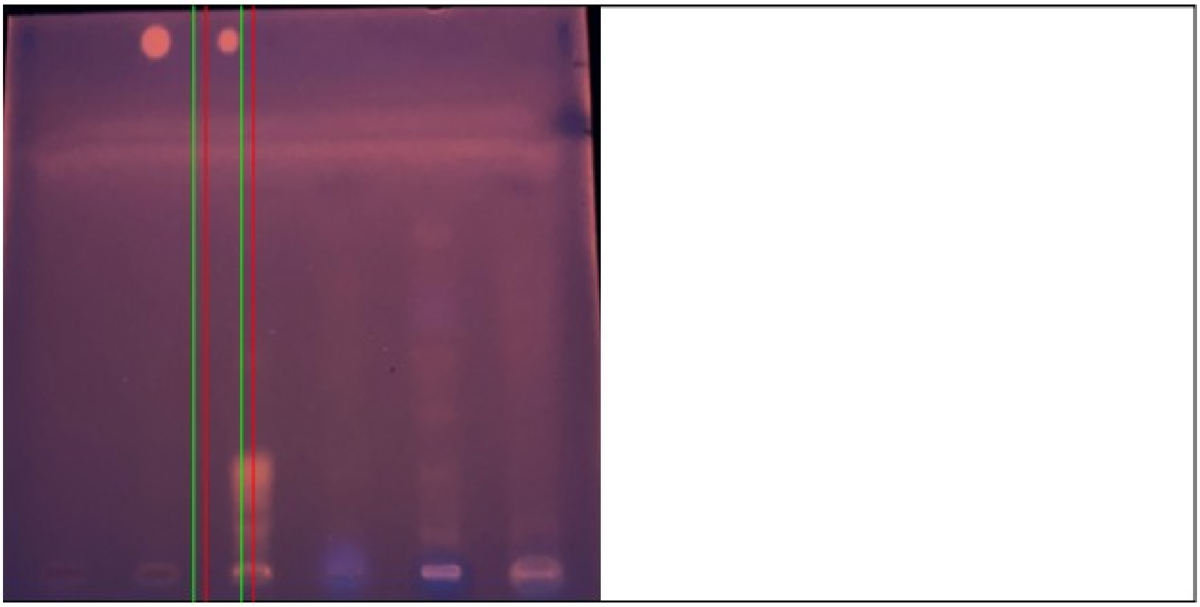

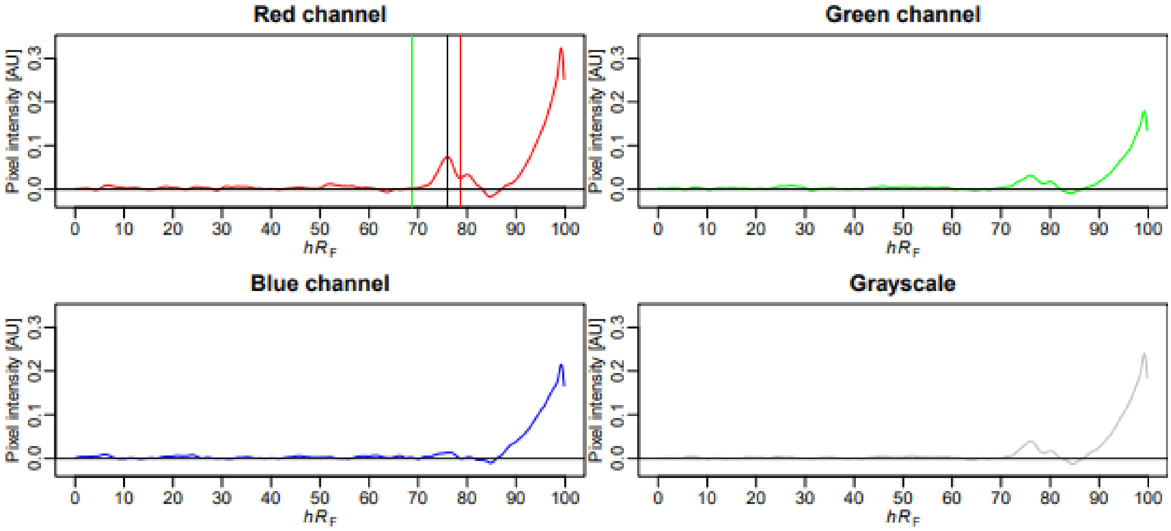

Track	Channel	Start hRf	End hRf	hRf	Height	Area
1	red	69	79	76	0.0752115	1.548056

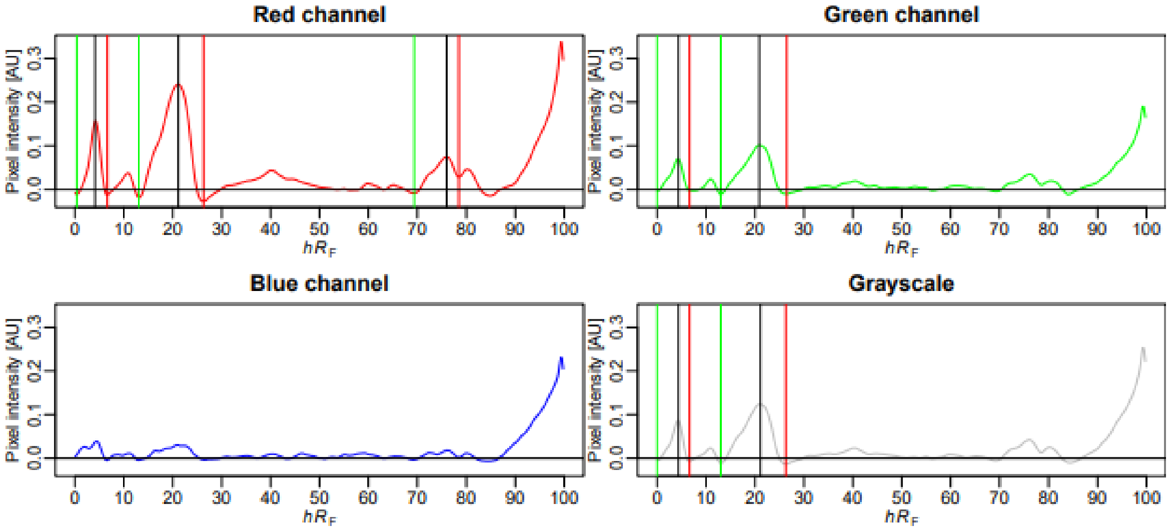

Track	Channel	Start hRf	End hRf	hRf	Height	Area
2	red	70	79	76	0.0744155	1.7559586
2	red	13	26	21	0.2413177	7.0413755
2	red	0	7	4	0.1568167	1.7054046
2	green	13	27	21	0.1013021	2.8592919
2	green	0	7	4	0.0706276	0.9242916
2	gray	13	26	21	0.1242581	3.6206965
2	gray	0	7	4	0.0873048	1.0375666

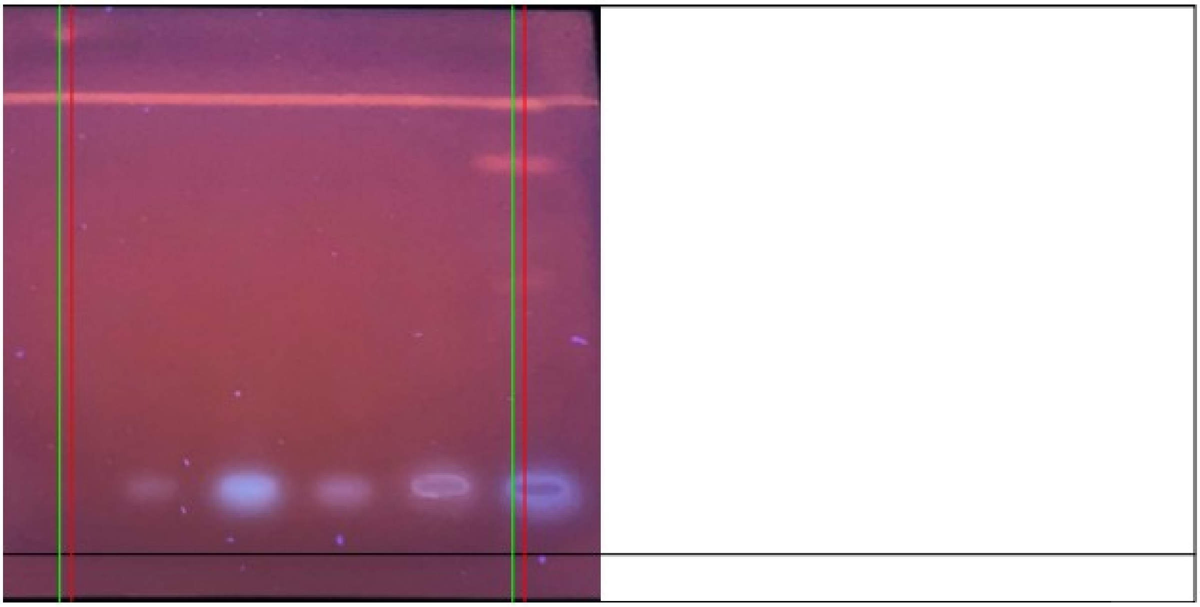

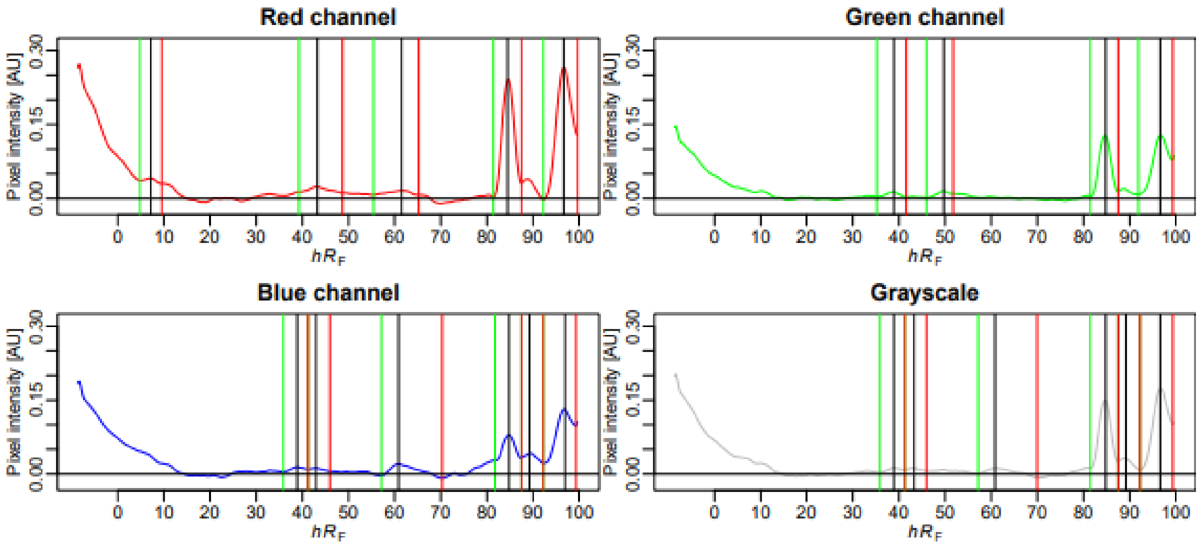

Track	Channel	Start hRf	End hRf	hRf	Height	Area
1	red	93	100	97	0.2666494	3.2856170
1	red	82	88	85	0.2411564	2.2310228
1	red	56	65	62	0.0160756	0.3467002
1	red	39	49	43	0.0243347	0.4997106
1	red	5	10	7	0.0411558	0.5465576
1	green	92	100	97	0.1282306	1.6250278
1	green	82	88	85	0.1278047	1.1682905
1	green	46	52	50	0.0148743	0.1607972
1	green	35	42	39	0.0135081	0.1508083
1	blue	93	100	97	0.1315707	1.8406572
1	blue	88	93	90	0.0421751	0.5114318
1	blue	82	88	85	0.0798698	0.9408340
1	blue	57	71	61	0.0206943	0.2832455
1	blue	41	46	43	0.0116091	0.1380459
1	blue	36	41	39	0.0141738	0.1701674
1	gray	93	100	97	0.1738947	2.1738363
1	gray	88	93	90	0.0321212	0.3420454
1	gray	82	88	85	0.1490837	1.4473649
1	gray	57	70	61	0.0114096	0.1653619
1	gray	41	46	43	0.0117663	0.1392587
1	gray	36	41	39	0.0124212	0.1449695

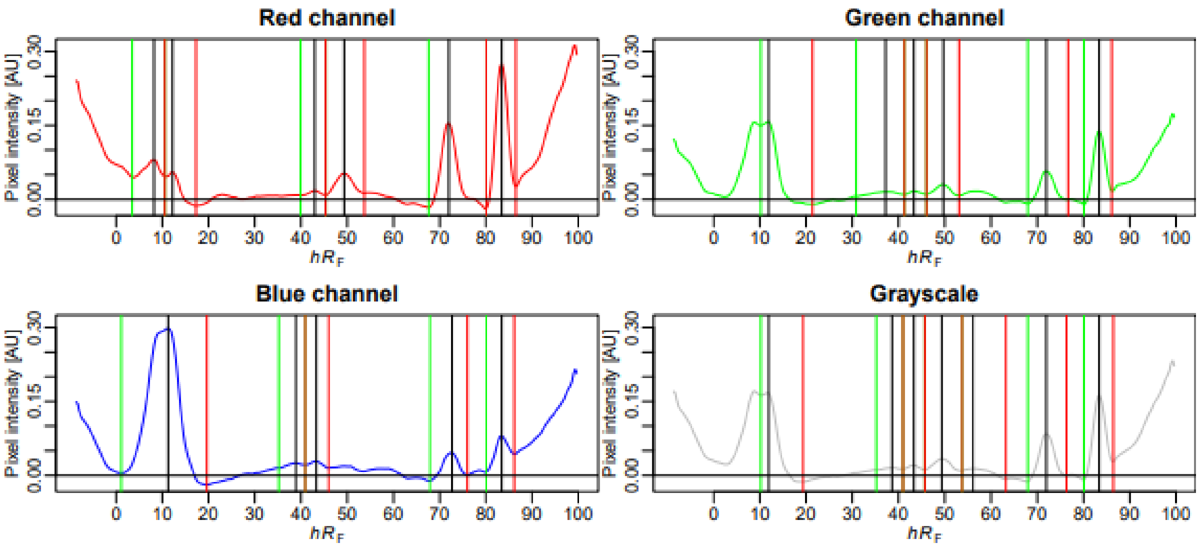

Track	Channel	Start hRf	End hRf	hRf	Height	Area
2	red	80	87	84	0.2755163	2.4543457
2	red	68	80	72	0.1548806	1.4960827
2	red	45	54	50	0.0525789	0.7854394
2	red	40	45	43	0.0177524	0.2125076
2	red	11	17	12	0.0552191	0.4314968
2	red	3	11	8	0.0799712	1.3297780
2	green	80	86	84	0.1387638	1.2066998
2	green	68	77	72	0.0569588	0.5566503
2	green	46	53	50	0.0298083	0.4121484
2	green	41	46	43	0.0168861	0.2019666
2	green	31	41	37	0.0155888	0.3838377
2	green	10	21	12	0.1582133	1.6469210
2	blue	80	86	84	0.0795270	0.9289524
2	blue	68	76	73	0.0461021	0.4464322
2	blue	41	46	43	0.0286194	0.3680547
2	blue	35	41	39	0.0251194	0.3821509
2	blue	1	20	11	0.2978597	6.6734002
2	gray	80	87	84	0.1631476	1.5218357
2	gray	68	77	72	0.0843883	0.8418809
2	gray	54	63	56	0.0141515	0.2157886
2	gray	46	54	50	0.0334776	0.5274403
2	gray	41	46	43	0.0206981	0.2459136
2	gray	35	41	39	0.0158322	0.2518813
2	gray	10	19	12	0.1670479	1.7881135

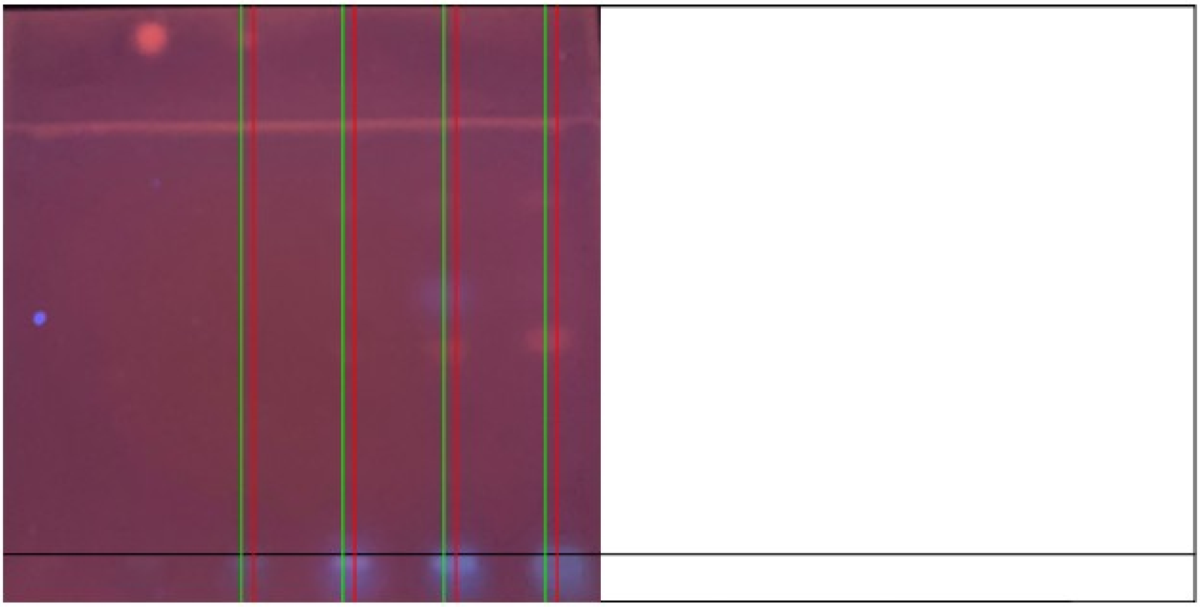

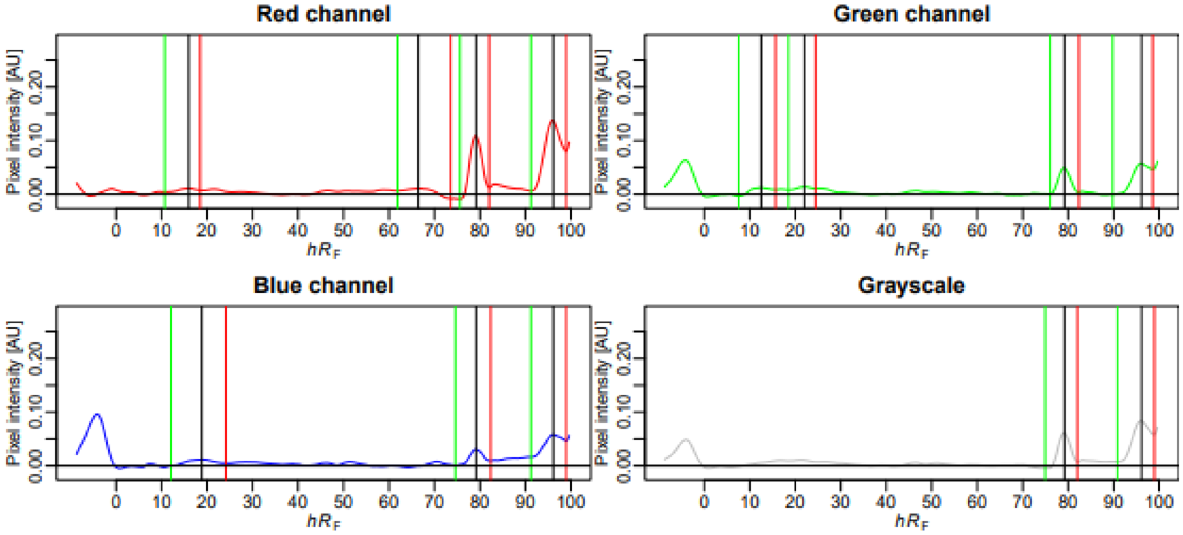

Track	Channel	Start hRf	End hRf	hRf	Height	Area
1	red	92	99	96	0.1374132	1.8020028
1	red	76	82	79	0.1090074	0.9237290
1	red	62	74	67	0.0108002	0.1727357
1	red	11	19	16	0.0111900	0.1805604
1	green	90	99	96	0.0574939	0.7518560
1	green	76	83	79	0.0491007	0.4385071
1	green	19	25	22	0.0138077	0.2041505
1	green	8	16	13	0.0123337	0.1724232
1	blue	92	99	96	0.0577061	0.9373256
1	blue	75	83	79	0.0303764	0.3254443
1	blue	12	24	19	0.0113444	0.2380355
1	gray	91	99	96	0.0841286	1.1827086
1	gray	75	82	79	0.0612446	0.5181359

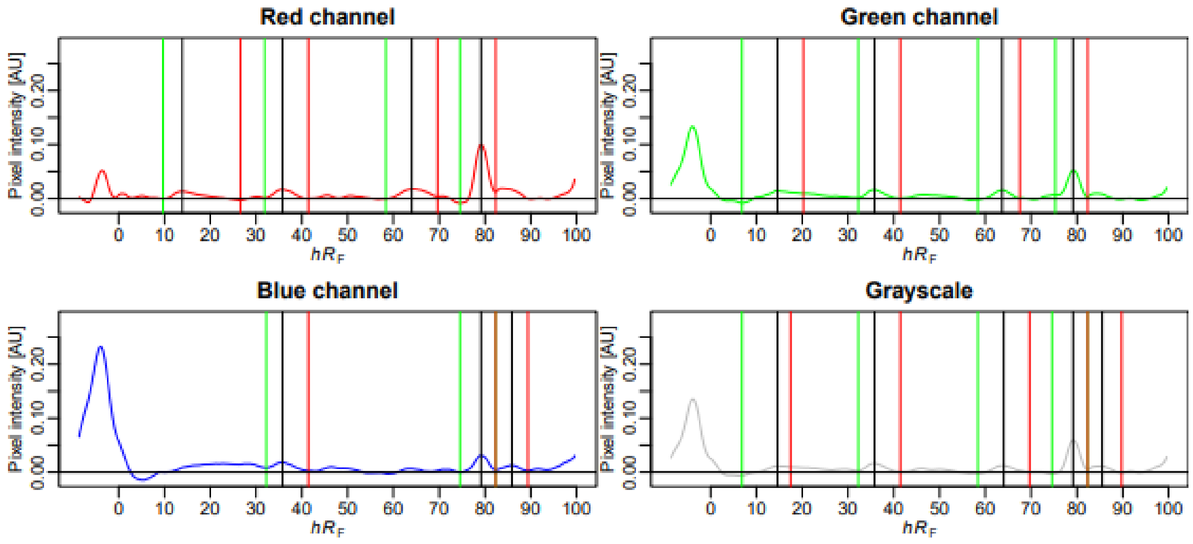

Track	Channel	Start hRf	End hRf	hRf	Height	Area
2	red	75	83	79	0.1001832	0.8761681
2	red	59	70	64	0.0178697	0.3359142
2	red	32	42	36	0.0171551	0.2347775
2	red	10	27	14	0.0132778	0.2370827
2	green	76	83	79	0.0523969	0.5141784
2	green	59	68	64	0.0156218	0.1782112
2	green	32	42	36	0.0164877	0.2126470
2	green	7	20	15	0.0144909	0.2576228
2	blue	83	90	86	0.0125929	0.1672222
2	blue	75	83	79	0.0311149	0.3192820
2	blue	32	42	36	0.0181958	0.2963933
2	gray	83	90	86	0.0112729	0.1464005
2	gray	75	83	79	0.0592802	0.5296984
2	gray	59	70	64	0.0122136	0.1605453
2	gray	32	42	36	0.0163792	0.2247995
2	gray	7	18	15	0.0113986	0.1359947

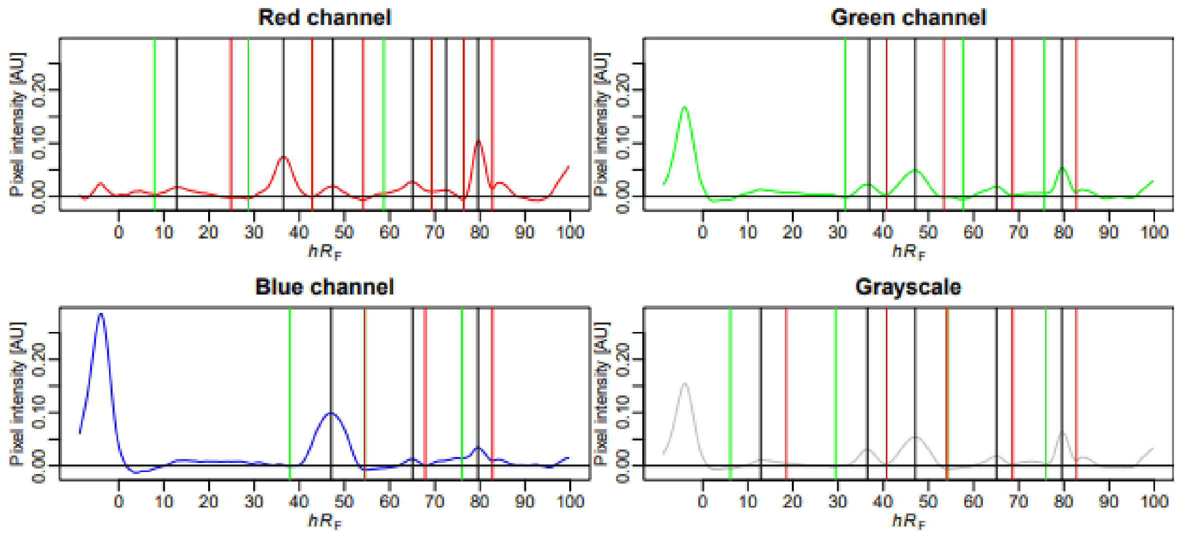

Track	Channel	Start hRf	End hRf	hRf	Height	Area
3	red	77	83	80	0.1041885	0.9201232
3	red	70	77	73	0.0117263	0.1335268
3	red	59	70	65	0.0271853	0.4648670
3	red	43	54	48	0.0191023	0.2183861
3	red	29	43	37	0.0748983	1.0575178
3	red	8	25	13	0.0174023	0.3580905
3	green	76	83	80	0.0534520	0.5249895
3	green	58	69	65	0.0186235	0.2370936
3	green	41	54	47	0.0491524	0.8891327
3	green	32	41	37	0.0223427	0.2887704
3	blue	76	83	80	0.0343389	0.4301185
3	blue	55	68	65	0.0126310	0.0197112
3	blue	38	55	47	0.0991320	2.1061768
3	gray	76	83	80	0.0631470	0.6043587
3	gray	54	69	65	0.0182906	0.1709378
3	gray	41	54	47	0.0539749	1.0088578
3	gray	30	41	37	0.0304493	0.3938354
3	gray	6	19	13	0.0111593	0.1627665

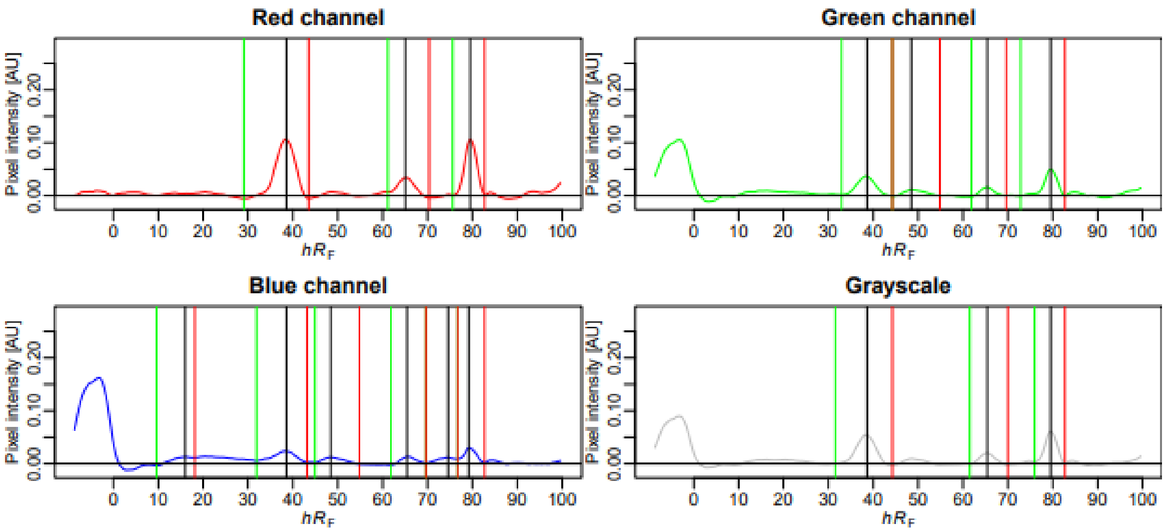

Track	Channel	Start hRf	End hRf	hRf	Height	Area
4	red	76	83	80	0.1066248	0.9555916
4	red	61	71	65	0.0345054	0.4159033
4	red	29	44	39	0.1062757	1.4396382
4	green	73	83	80	0.0500122	0.4739663
4	green	62	70	66	0.0162461	0.1487306
4	green	44	55	49	0.0115212	0.1627612
4	green	33	44	39	0.0370813	0.5012343
4	blue	77	83	79	0.0294593	0.2844554
4	blue	70	77	75	0.0118409	0.1579381
4	blue	62	70	66	0.0131873	0.1327934
4	blue	45	55	49	0.0122447	0.1803666
4	blue	32	43	39	0.0238535	0.4310508
4	blue	10	18	16	0.0130093	0.1772397
4	gray	76	83	80	0.0611079	0.5520677
4	gray	62	70	66	0.0202454	0.2047759
4	gray	32	44	39	0.0540730	0.7408919
